# Individual Factors Influencing the Public’s Perceptions About the Importance of COVID-19 Immunity Certificates in the United Kingdom: Cross-sectional Web-based Questionnaire Survey

**DOI:** 10.2196/37139

**Published:** 2023-04-27

**Authors:** Corina-Elena Niculaescu, Isabel Karen Sassoon, Irma Cecilia Landa-Avila, Ozlem Colak, Gyuchan Thomas Jun, Panagiotis Balatsoukas

**Affiliations:** 1 Department of Computer Science Brunel University London London United Kingdom; 2 School of Design and Creative Arts Loughborough University Loughborough United Kingdom

**Keywords:** immunity passports, immunity certificates, vaccine passports, COVID-19, health belief model, vaccination, pandemic, cross-sectional survey, low income, vulnerable population, socioeconomic, public perception, public policy

## Abstract

**Background:**

Understanding how perceptions around immunity certificates are influenced by individual characteristics is important to inform evidence-based policy making and implementation strategies for services around immunity and vaccine certification.

**Objective:**

This study aimed to assess what were the main individual factors influencing people’s perception of the importance of using COVID-19 immunity certificates, including health beliefs about COVID-19, vaccination views, sociodemographics, and lifestyle factors.

**Methods:**

A cross-sectional web-based survey with a nationally representative sample in the United Kingdom was conducted on August 3, 2021. Responses were collected and analyzed from 534 participants, aged 18 years and older, who were residents of the United Kingdom. The primary outcome measure (dependent variable) was the participants’ perceived importance of using immunity certificates, computed as an index of 6 items. The following individual drivers were used as the independent variables: (1) personal beliefs about COVID-19 (using constructs adapted from the Health Belief Model), (2) personal views on vaccination, (3) willingness to share immunity status with service providers, and (4) variables related to respondents’ lifestyle and sociodemographic characteristics.

**Results:**

The perceived importance of immunity certificates was higher among respondents who felt that contracting COVID-19 would have a severe negative impact on their health (β=0.2564; *P*<.001) and felt safer if vaccinated (β=0.1552; *P*<.001). The prospect of future economic recovery positively influenced the perceived importance of immunity certificates. Respondents who were employed or self-employed (β=–0.2412; *P*=.001) or experienced an increase in income after the COVID-19 pandemic (β=–0.1287; *P*=.002) perceived the use of immunity certificates as less important compared to those who were unemployed or had retired or those who had experienced a reduction in their income during the pandemic.

**Conclusions:**

The findings of our survey suggest that more vulnerable members in our society (those unemployed or retired and those who believe that COVID-19 would have a severe impact on their health) and people who experienced a reduction in income during the pandemic perceived the severity of not using immunity certificates in their daily life as higher.

## Introduction

Although quite a few studies have tried to explore the role of different individual characteristics on attitudes toward vaccination [1-4], there is little known about their role on people’s attitudes toward immunity certificates. The term “immunity certificate” is defined as evidence (in digital or paper format) that an individual has developed antibodies of SARS-CoV-2 through past infection or vaccination [5,6]. Immunity certificates, and their terminological variation such as immunity passports or vaccine passports, have been at the center of controversy as their value polarizes opinions among academics, policy makers, and the general public. Both perceived benefits of and concerns about immunity certificates have been reported in the literature. For example, preserving the freedom of movement [7] and reopening the economy and reducing the risk of infection [8,9] are some frequently reported benefits, whereas the loss of autonomy [10-15], legal challenges [16,17], the risk of fraud [12], and digital exclusion [8,18,19] represent some of the most prominent concerns. This knowledge is useful to understand the drivers and barriers of implementing immunity certificates in general. However, empirical evidence is needed to understand how different individual factors and characteristics may influence the prevalence of those drivers or barriers. The production of this knowledge is important to help us understand how perceptions around immunity certificates are influenced by individual characteristics and use this insight to inform policy making and implementation strategies for services around immunity certification, for example, by helping identify those who are more in need of using immunity certificates [5,6].

The aim of this paper was to report the findings of a UK-wide, web-based questionnaire survey assessing the role of different individual factors on the perceived importance of using immunity certificates. Specifically, we examined the following types of individual factors: personal beliefs about COVID-19, views on vaccination, willingness to share their immunity status, lifestyle, and sociodemographic characteristics. Throughout this paper, we use the term “immunity certificate” to describe a service that allows individuals with antibodies of SARS-CoV-2, obtained through past infection or after a full course of vaccination, to evidence their immunity status.

## Methods

### Sample Design

Our analysis is based on a cross-sectional data set obtained from a web-based anonymous questionnaire survey, designed using the web-based platform Online Surveys [20]. Responses were collected using Prolific [21] on August 3, 2021. Respondents were demographically representative of the UK population in terms of gender, age, and ethnicity. We excluded 20 participants who failed the attention checks and 1 duplicate responder, resulting in a final sample of 534 respondents. All participants were aged 18 years or older and were compensated for their participation in the study with £1.75 (US $2.15) per person. All materials including data set, statistical codes, questionnaire survey, and ethics approval can be accessed on Open Science Framework [22].

The sample size was estimated using Vaske [23] and a pragmatic range for the assumptions. The estimate for sample size ranged from 271 and 1067 participants, depending on the assumptions. The final sample size falls within this range.

### Main Variables Measure—Perceived Importance of Using Immunity Certificates (Primary Outcome)

**Figure 1:**
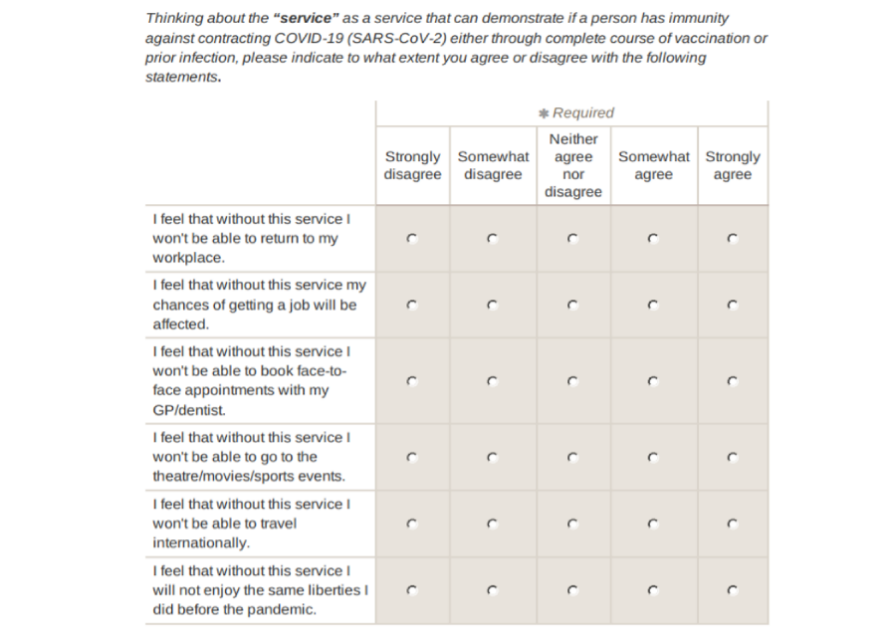
Screenshot of the survey questions on the perceived severity of using immunity certificates [22]. GP: general practitioner.

The perceived importance of using immunity certificates was the computed index of 6 items, each measuring a different area where the use of immunity certificates could impact people’s lives. A screenshot of the 6 items used is presented in Figure 1. Table 1 presents summary statistics for all variables used. These 6 items were informed by the findings of a series of focus groups and interviews investigating the public’s concerns about the risks and unintended consequences of immunity certificates [5]. Responses to these items were measured on a 5-point Likert scale from (1=“Strongly disagree” to 5=“Strongly agree”).

**Figure 2:**
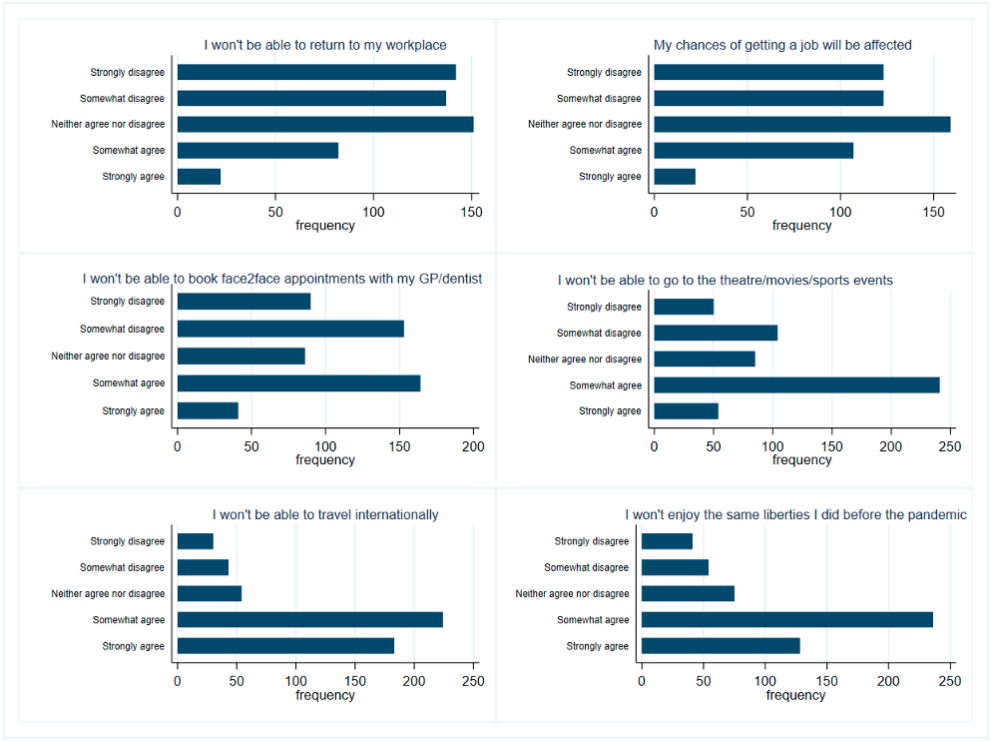
Distribution of responses across perceived severity of using immunity certificates [22]. GP: general practitioner.

The distribution of responses for each item is presented in Figure 2. Subsequently, we observed that the internal reliability of the 6 items was high (0.8485; Table 1) [24]. Therefore, we measured the overall perceived importance of using immunity certificates by creating the index *Certificate Severity*. This index was computed as the average score among its 6 component items, and it is a continuous variable taking value between 1 and 5 [25].

**Table 1:** Summary statistics of Health Belief Model (HBM) measures, vaccine views, lifestyle variables, and willingness to share immunity status with service providers.

HBM measures and items		Mean (SD)	Median (range)	Cronbach α	
**Perceived importance of using immunity certificates**					0.8485
	I feel that without this service I won’t be able to return to my workplace.	2.4476 (1.1558)	2 (1-5)		
	I feel that without this service my chances of getting a job will be affected.	2.5918 (1.1631)	3 (1-5)		
	I feel that without this service I won’t be able to book face-to-face appointments with my GP^a^/dentist.	2.8371 (1.2455)	3 (1-5)		
	I feel that without this service I won’t be able to go to the theatre/movies/sports events.	3.2715 (1.1636)	4 (1-5)		
	I feel that without this service I won’t be able to travel internationally.	3.912 (1.1252)	4 (1-5)		
	I feel that without this service I will not enjoy the same liberties I did before the pandemic.	3.6667 (1.1692)	4 (1-5)		
**Perceived COVID-19 susceptibility**					0.7095
	I am at risk of getting COVID-19 (SARS-CoV-2).	3.5243 (1.1255)	4 (1-5)		
	It is likely that I will get COVID-19 (SARS-CoV-2).	2.9401 (1.0122)	3 (1-5)		
	Individuals in my household are at risk for getting COVID-19 (SARS-COV-2).	3.4438 (1.131)	4 (1-5)		
	I feel knowledgeable about my risk of getting COVID-19 (SARS-COV-2).	4.1255 (0.746)	4 (1-5)		
**Perceived COVID-19 severity**					0.7061
	I believe that COVID-19 (SARS-CoV-2) is a severe health problem in general.	4.2266 (0.9662)	4 (1-5)		
	If I get COVID-19 (SARS-CoV-2) I will get sick.	3.7247 (0.9749)	4 (1-5)		
	If I get COVID-19 (SARS-CoV-2) I will die.	2.1386 (0.9227)	2 (1-5)		
	If I get COVID-19 (SARS-CoV-2) other members in my household will get sick.	3.5824 (1.012)	4 (1-5)		
**Perceived benefits of immunity certificates**					0.6045
	This service will make me feel safe only if immunity is obtained through complete course of vaccination.	3.2809 (1.141)	3 (1-5)		
	This service will make me feel safe only if immunity is obtained through past COVID-19 (SARS-CoV-2) infection.	2.4326 (0.9734)	2 (1-5)		
	This service will facilitate economic recovery.	3.5506 (1.054)	4 (1-5)		
	This service will facilitate social gatherings in closed spaces without restrictions (e.g. wearing masks, limits on number of people who can gather).	3.7154 (0.9922)	4 (1-5)		
**Perceived barriers of using immunity certificates**					0.3691
	I'm afraid that my data will be passed on to third parties without my consent or commercialized.	3.0281 (1.2883)	3 (1-5)		
	This service will be difficult for me to use if available only on smartphones/tablets.	1.9307 (1.1913)	2 (1-5)		
	This service will be difficult for me to access if offered exclusively in English.	1.2809 (0.6982)	1 (1-5)		
**Hopelessness after COVID-19**					
	Mental wellbeing after COVID-19	2.6685 (0.7606)	3 (1-5)		
	Net income after COVID-19	2.8221 (0.8445)	3 (1-5)		
**Vaccine views**					
	I am not convinced that the vaccine will protect me against COVID-19 (SARS-CoV-2).	2.3034 (1.2212)	2 (1-5)		
	I feel worried about people who have received a non-UK approved vaccine entering the country.	2.6292 (1.2055)	3 (1-5)		
**Lifestyle**					
	Travel internationally for business	1.382 (0.7428)	1 (1-4)		
	Travel internationally for leisure	2.633 (0.923)	3 (1-4)		
	Travel internationally to visit family and/or friends	2.0243 (1.0293)	2 (1-4)		
	Book accommodation (hotels, Airbnb etc.)	2.8333 (0.8879)	3 (1-4)		
	Attend sports events	2.03 (0.9583)	2 (1-4)		
	Go to the theatre or movies	2.8015 (0.8521)	3 (1-4)		
	Visit museums, galleries and other cultural exhibitions or festivals	2.7828 (0.809)	3 (1-4)		
	Go to a pub, restaurant, club or coffee shop for a meal or drink.	3.4045 (0.7634)	4 (1-4)		
	Care for or visit someone who lives in a care home.	1.5112 (0.885)	1 (1-4)		
**Willingness to share immunity status with service providers**					
	Theatre/cinema/gallery	3.2921 (1.3998)	4 (1-5)		
	Pub/restaurant	3.2228 (1.4159)	4 (1-5)		
	GP/dentist	4.47 (0.9219)	5 (1-5)		
	Hospitality sector	3.4663 (1.3717)	4 (1-5)		
	Sports event	3.3015 (1.4012)	4 (1-5)		
	Airport/airline	3.8764 (1.2538)	4 (1-5)		

^a^GP: general practitioner.

### Independent Variables

#### Personal Beliefs About COVID-19

We measured respondents’ personal beliefs about COVID-19 using 4 constructs adapted from the Health Belief Model [26] and tailored to the needs of this study. The detailed description of the items, summary statistics, and internal reliability measures are presented in Table 1. Each item was rated on a 5-point Likert scale from 1 (“Strongly disagree”) to 5 (“Strongly agree”). First, we measured perceived COVID-19 susceptibility using 3 items adapted from Coe et al [2] and 1 item from Chu and Liu [27]. Second, we measured perceived COVID-19 severity through 4 items adapted from Coe et al [2]. Perceived COVID-19 susceptibility measures respondents’ perceived risk of contracting the SARS-CoV-2 virus, whereas perceived COVID-19 severity represents the perceived severity of negative health consequences if the respondent were to contract the virus. Third, we measured perceived barriers from using immunity certificates with 3 items referring to data safety and accessibility (smartphone availability and language). Finally, we measured perceived benefits of using immunity certificates through 4 items covering safety, economic recovery, and return to social gatherings.

As presented in Table 1, perceived COVID-19 susceptibility and perceived COVID-19 severity display a Cronbach α of 0.7 or higher, suggesting good internal consistency. Therefore, we created an index for each of these constructs (*Perceived COVID-19 Susceptibility* and *Perceived COVID-19 Severity*) by averaging the items within the constructs [28,29]. For perceived barriers and perceived benefits of using immunity certificates, we used the individual items in our analysis, without transforming these into indices, as their Cronbach α was lower than 0.7 [24].

#### Vaccination Views

At the time when our study was conducted, approximately 75% of the UK’s adult population had been vaccinated [30]. Therefore, instead of using the traditional Health Belief Model constructs of measuring intention to get vaccinated, vaccination barriers, or perceived severity of COVID-19 vaccines, we asked 3 questions on vaccination views that our previous qualitative research showed were common concerns among both fully vaccinated and not vaccinated individuals [5]. As such, we constructed 3 questions about respondents’ perceived vaccine effectiveness, worries about non–UK-approved vaccines, and feeling of safety around vaccinated people. Each item was rated on a 5-point Likert scale from 1 (“Strongly disagree”) to 5 (“Strongly agree”).

#### Lifestyle Prior to COVID-19

We asked a series of lifestyle-related questions to determine if respondents’ habits before the COVID-19 outbreak had an effect, if any, on the primary outcome measure. Lifestyle questions measured the frequency with which respondents engaged with a series of social activities using a 4-point Likert scale ranging from 1 (“Never”) to 4 (“Very often”). The complete list of questions is presented in Table 1. In summary, these measured the frequency with which respondents travelled internationally; booked accommodation when travelling; attended sports events; went to theatres/movies or visited other cultural events; went to pubs, restaurants, and other dinning venues; or visited a health care setting (eg, visited someone in a care home). Similar to questions about vaccination views, the lifestyle questions were informed by the findings of our qualitative research conducted between February and July 2021 [5].

#### Willingness to Share Immunity Status With Different Service Providers

Respondents were asked to rate their level of agreement in sharing their immunity status with different service providers on a 5-point Likert scale ranging from 1 (“Strongly disagree”) to 5 (“Strongly agree”). The types of service providers for which respondents had to rate their level of agreement included their general practitioner or dentist; airport or airline; hospitality sector (eg, hotels and other booked accommodation); theatre, cinema, or gallery; sports event; and pub, restaurant, or nightclub.

#### Sociodemographics

Summary statistics for the sociodemographic variables used in this study are presented in Table 2. In addition to the representative gender, age, and ethnicity variables, we also recorded data about respondents’ geographic location in the United Kingdom (urban or rural), accommodation arrangements (eg, living alone or in shared accommodation), employment status, education, and whether or not the respondent had a disability.

The COVID-19 pandemic and subsequent lockdown measures have been difficult for many people, leading to deceased mental well-being [31-36], unemployment, and lower income [37,38]. Therefore, to control for the possibility of attitudes toward the primary outcome measure streaming from feelings of hopelessness, we measured perceived mental well-being and net income now compared to before the beginning of the pandemic using a 5-point Likert scale ranging from 1 (“Much worse” or ”Much lower”) to 5 (“Much better” or ”Much higher”).

**Table 2:** Demographic characteristics of sample.

Demographic characteristic		Respondents (N=543), n (%)	Cumulative percentage (%)
**Gender**			
	Female	277 (51.9)	51.9
	Male	254 (47.6)	99.4
	Prefer not to say	2 (0.4)	99.8
	Self-defined	1 (0.2)	100
**Age range (years)**			
	18-23	77 (14.4)	14.4
	24-29	51 (9.6)	24
	30-39	95 (17.8)	41.8
	40-49	87 (16.3)	58.1
	50-59	95 (17.8)	75.8
	60-69	109 (20.4)	96.3
	70 or older	20 (3.7)	100
**Race/ethnicity**			
	Asian	34 (6.4)	6.4
	Black	20 (3.7)	10.1
	Hispanic/Latino	3 (0.6)	10.7
	Mixed	15 (2.8)	13.5
	Other	8 (1.5)	15
	South Asian	12 (2.2)	17.2
	White	442 (82.8)	100
**Region**			
	East Midlands	42 (7.9)	7.9
	East of England	35 (6.6)	14.4
	London	81 (15.2)	29.6
	Northeast	32 (6)	35.6
	Northern Ireland	11 (2.1)	37.6
	Northwest England	58 (10.9)	48.5
	Scotland	37 (6.9)	55.4
	South-East England	87 (16.3)	71.7
	Southwest of England	43 (8.1)	79.8
	Wales	19 (3.6)	83.3
	West Midlands	45 (8.4)	91.8
	Yorkshire and the Humber	44 (8.2)	100
**Area**			
	Rural	166 (31.1)	31.1
	Urban	368 (68.9)	100
**Accommodation**			
	Living alone	87 (16.3)	16.3
	Living in shared accommodation	54 (10.1)	26.4
	Living with other family members	382 (71.5)	97.9
	Other	11 (2.1)	100
**Employment**			
	Employed or self-employed	340 (63.7)	63.7
	Retired	97 (18.2)	81.8
	Unemployed	97 (18.2)	100
**Education**			
	A level^a^ (or equivalent)	130 (24.3)	24.3
	GCSE^b^ (or equivalent)	80 (15)	39.3
	Postgraduate degree	95 (17.8)	57.1
	Undergraduate degree	175 (32.8)	89.9
	Vocational	54 (10.1)	100
**Disability**			
	No	467 (87.5)	87.5
	Prefer not to say	6 (1.1)	88.6
	Yes	61 (11.4)	100

^a^A level: advanced level.

^b^GCSE: General Certificate of Secondary Education.

### Statistical Analysis

**Figure 3:**
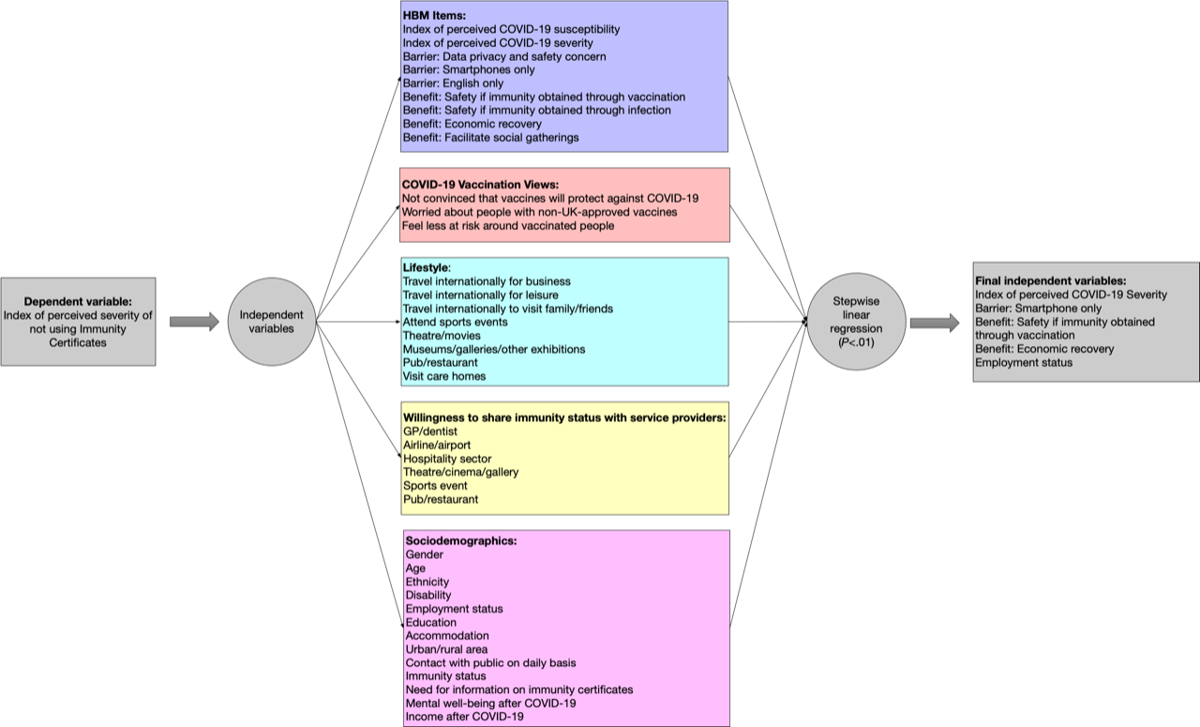
Illustration of the statistical analysis [22]. DV: dependent variable; GP: general practitioner; HBM: Health Belief Model.

To address our research questions, we used a multiple stepwise linear regression analysis using *Certificate Severity* (ie, respondents’ perceived importance of using immunity certificates) as the dependent variable and the independent variables described above. *P* values smaller than .01 were used as the threshold to indicate the significance of the estimated coefficients. This analysis was performed in Stata software (version 17; StataCorp) [39]. Stepwise regression analysis was used in other studies exploring COVID-19 vaccination views [40,41], relationships between a COVID-19 risk index and COVID-19 mortality rates [42], and anxiety and depression during COVID-19 [31]. A graphical representation of the steps used in our statistical analysis is presented in Figure 3.

### Power Calculation

The sample size was chosen pragmatically based on several different approaches [43], obtaining a minimum sample size between 271 and 1067 participants, depending on the assumptions.

### Ethics Approval

Ethics approval was obtained from the College of Engineering, Design and Physical Sciences Research Ethics Committee at Brunel University London (Ref. 31705-A-Jul/2021-33586-1) on July 29, 2021. Informed consent was obtained from all respondents prior to the beginning of the survey. Respondents were allowed to withdraw from the survey at any time.

## Results

**Figure 4:**
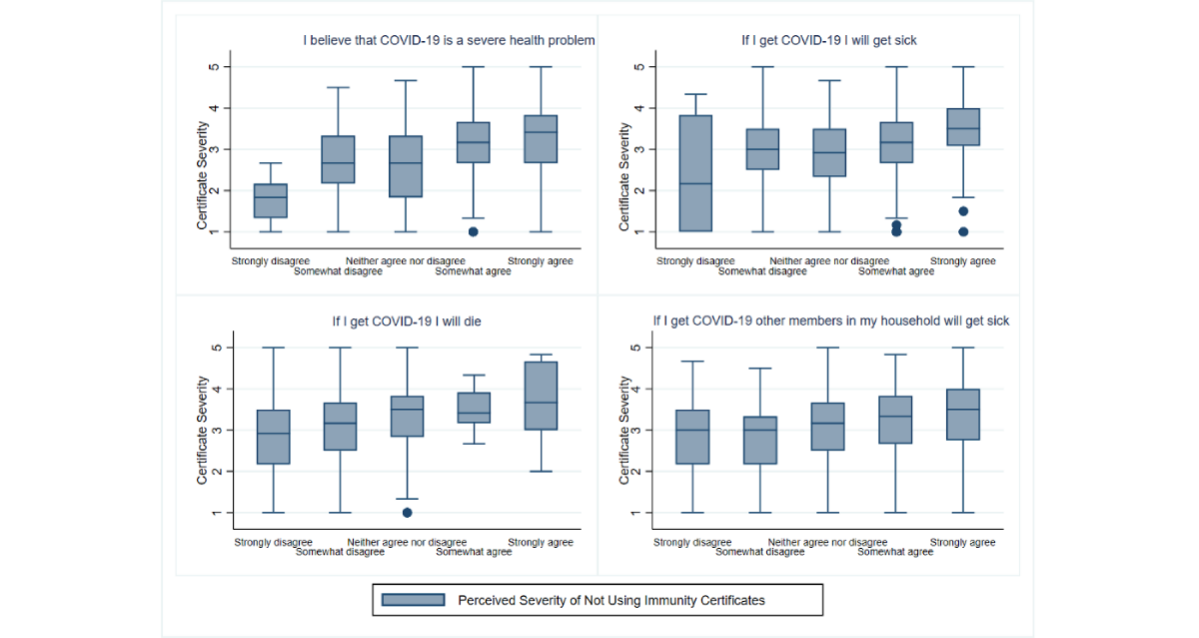
Perceived importance of using immunity certificates (certificate severity) by perceived COVID-19 severity.

Table 3 presents our statistical model after conducting the multiple stepwise linear regression analysis with *P*<.01. Respondents who perceived themselves as being more at risk of experiencing negative health consequences if they contracted the virus (Perceived COVID-19 Severity) were more likely to value positively the importance of immunity certificates (Certificate Severity), demonstrated with an increase of 0.2506 units (Table 3). Figure 4 illustrates the relationship between the perceived importance of using immunity certificates and Perceived COVID-19 Severity.

**Figure 5:**
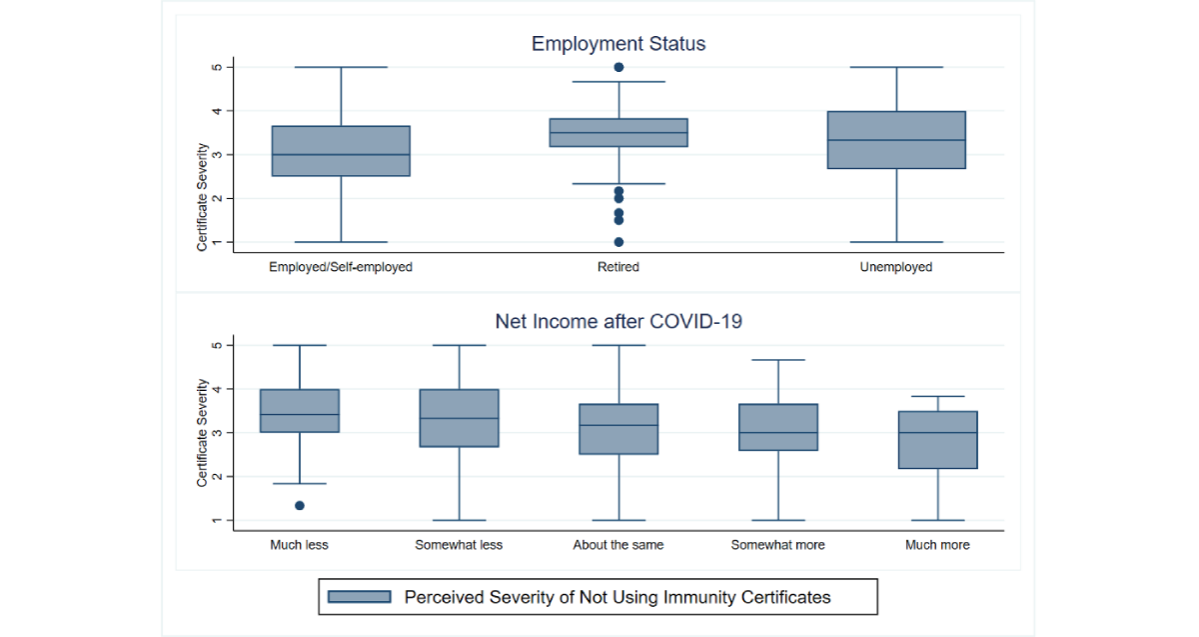
Perceived importance of using immunity certificates (certificate severity) by employment status and net income after COVID-19.

Similarly, those who felt safer if vaccinated and believed in the prospect of future economic recovery were more likely to perceive the use of immunity certificates as more important, demonstrated with an increase of 0.1594 and 0.1585 units in *Certificate Severity*, respectively (Table 3). Additionally, the results showed that those who were employed or self-employed or had experienced an increase in their net income after the COVID-19 outbreak were more likely to perceive the use of immunity certificates as less important. Specifically, compared to respondents who were retired or unemployed, those who were in employment (employed or self-employed) displayed a lower perceived importance of using immunity certificates (*Certificate Severity*) by 0.2343 units. The same negative effect was observed for people who reported higher levels of net income after the COVID-19 outbreak with a decrease of 0.1280 units in *Certificate Severity.* The relationship between the perceived importance of using immunity certificates, employment status, and net income after COVID-19 is presented in Figure 5.

Finally, the remaining independent variables used in the statistical analysis including *Perceived COVID-19 Susceptibility*, lifestyle, age, gender, and ethnicity (among others) did not have a statistically significant effect on the perceived importance of using immunity certificates.

**Table 3:** Stepwise linear regression results of certificate severity and perceived COVID-19 severity, benefit: safe if immunity obtained through vaccination, benefit: economic recovery, employed or self-employed, and income after COVID-19^a^.

Variable	β (SE)	95% CI	2-tailed *t* test (*df*)^b^	*P* value
Perceived COVID-19 severity	0.2506 (0.0505)	0.1513 to 0.3498	4.9600	<.001
Benefit: safe if immunity obtained through vaccination	0.1594 (0.0325)	0.0955 to 0.2233	4.9000	<.001
Benefit: economic recovery	0.1585 (0.0344)	0.0909 to 0.2261	4.6100	<.001
Employed or self-employed	–0.2343 (0.0715)	–0.3747 to –0.0939	–3.2800	.001
Income after COVID-19	–0.1280 (0.0408)	–0.2082 to –0.0478	–3.1400	.002
(Constant)	1.6911(0.2292)	1.2408 to 2.1414	7.3800	<.001

^a^The adjusted *R*^2^ of this regression is 22.76%. Employed or self-employed is a dummy variable that equals 1 if the respondent was either employed or self-employed at the time of the survey and 0 if they are either retired or unemployed.

^b^The degree of freedom (*df*) of this regression model is 520.

## Discussion

### Principal Findings

The findings of our research suggest that people who are more vulnerable (not working and believing that contracting COVID-19 would have a severe impact on their health) are more responsive to the use of immunity certificates, and therefore, the importance of using them in daily life is perceived as higher. Additionally, respondents perceived the importance of immunity certificates as higher if immunity was acquired after a full course of vaccination compared to past infection. These findings partially confirm the results of previous studies where the authors investigated the role of personal health beliefs on vaccination [1-3]. Moreover, as opposed to previous research on attitudes toward vaccination, we did not find an effect of age, gender, and ethnic background when it comes to the perceived importance of immunity certificates [1,2,27]. However, we did observe a significant effect of employment status and loss of income, suggesting the importance of socioeconomic factors compared to demographics in this context.

### Limitations

One of the limitations of our study is that participants were recruited from the web-based survey platform Prolific. Since Prolific surveys are completed digitally (mobile, PC, tablet, etc), our sample was comprised of people who had the means and capacity to use digital technologies.

Another limitation of our study is the relatively low explanatory power of our model with an adjusted *R*^2^ of 22.76%, suggesting that the independent variables chosen by our stepwise linear regression model only explains 22.76% of the observed variation in the index *Certificate Severity.* Considering that research on immunity certificates is still in its early stages, we do not yet have a large body of literature to draw from to identify more predictors of *Certificate Severity.* More research is needed to explore what the factors that we did not capture could be.

### Conclusions

Understanding the role of individual factors on the perceived importance of immunity certificates is necessary to make evidence-based decisions when considering their design and implementation. Such decisions should aim to protect vulnerable members of our society and those in need.

## Data Availability

The data is available in a public, open access repository. All materials are freely available on Open Science Framework [22].
